# Histone deacetylase inhibitors suppress aggressiveness of head and neck squamous cell carcinoma via histone acetylation-independent blockade of the EGFR-Arf1 axis

**DOI:** 10.1186/s13046-019-1080-8

**Published:** 2019-02-18

**Authors:** Leilei He, Lixia Gao, Chloe Shay, Liwei Lang, Fenglin Lv, Yong Teng

**Affiliations:** 1 College of Bioengineering, Chongqing University, Chongqing, 400044 People’s Republic of China; 2 Department of Oral Biology and Diagnostic Sciences, Dental College of Georgia, Augusta University, Augusta, GA 30912 USA; 3 Department of Pediatrics, School of Medicine, Emory University, Atlanta, GA USA; 4 Georgia Cancer Center, Department of Biochemistry and Molecular Biology, Medical College of Georgia, Augusta University, Augusta, GA USA; 5 Department of Medical Laboratory, Imaging and Radiologic Sciences, College of Allied Health, Augusta University, Augusta, GA USA; 6 Chongqing University of Arts and Sciences, Chongqing, 402160 People’s Republic of China

**Keywords:** HDAC, Arf1, EGFR, HNSCC, Invasion, Anticancer

## Abstract

**Background:**

A promising arsenal of histone deacetylase (HDAC)-targeted treatment has emerged in the past decade, as the abnormal targeting or retention of HDACs to DNA regulatory regions often occurs in many cancers. Head and neck squamous cell carcinoma (HNSCC) is one of the most aggressive malignancies worldwide associated with poor overall survival in late-stage patients. HDAC inhibitors have great potential to treat this devastating disease; however, few has been studied regarding the beneficial role of HDAC inhibition in anti-HNSCC therapy and the underlying molecular mechanisms remain elusive.

**Methods:**

Cell migration and invasion were examined by wound closure and Transwell assays. Protein levels and interactions were assessed by Western blotting and immunoprecipitation. HDAC activity was measured with the fluorometric HDAC Activity Assay. Phospho-receptor tyrosine kinase (RTK) profiling was determined by the Proteome Profiler Human Phospho-RTK Array.

**Results:**

ADP-ribosylation factor 1 (Arf1), a small GTPase coordinating vesicle-mediated intracellular trafficking, can be inactivated by HDAC inhibitors through histone acetylation-independent degradation of epidermal growth factor receptor (EGFR) in HNSCC cells. Mechanistically, high levels of Arf1 activity are maintained by binding to phosphorylated EGFR which is localized on HNSCC cell plasma membrane. Decreased EGFR phosphorylation is associated with reduced EGFR protein levels in the presence of TSA, which inactivates Arf1 and eventually inhibits invasion in HNSCC cells.

**Conclusions:**

Our insights explore the critical role of EGFR-Arf1 complex in driving HNSCC progression, and demonstrate the selective action of HDAC inhibitors on this specific axis for suppressing HNSCC invasion. This novel finding represents the first example of modulating the EGFR-Arf1 complex in HNSCC by small molecule agents.

**Electronic supplementary material:**

The online version of this article (10.1186/s13046-019-1080-8) contains supplementary material, which is available to authorized users.

## Background

Epigenetic alterations, including the reversible histone acetylation and deacetylation, contribute to the development and progression of human cancers by the modulation of chromatin topology and the regulation of gene expression [1, 2]. Histone deacetylases (HDACs) are part of a vast family of enzymes that remove the acetyl group from histone proteins on DNA, making the DNA less accessible to transcription factors. Based on sequence homology to yeast, eighteen human HDACs are grouped into four main classes: class I (HDACs 1, 2, 3, and 8), class II (HDACs 4, 5, 6, 7, and 9), class III (sirtuins) and class IV (contains only HDAC11 which shares sequences similarity to both class I and II proteins) [3, 4]. These enzymes are vital regulators of fundamental cellular events, such as cell cycle progression and differentiation, and have been found to dysregulate and/or function incorrectly in cancer [5]. The crucial roles of HDACs in numerous tumor activities, such as cell proliferation, cell-cycle regulation and apoptosis, are largely through their repressive influence on gene transcription, and the mechanisms by which individual HDACs regulate oncogenesis are quite diverse [5, 6]. HDACs are considered as promising drug targets to combat cancer; however, their regulatory network and molecular mechanisms in cancer progression, especially for cancer invasion and metastasis, remain to be clarified.

Histone deacetylase inhibitors, the compounds that interfere with the function of HDAC and lead to accumulation of acetylated nuclear histones, are potent anticancer agents with moderately little effect on normal tissues [7, 8]. Among them, SAHA (vorinostat) and PXD101 (belinostat) are pan-HDAC inhibitors developed for cancer treatment, whereas trichostatin A (TSA) is an organic compound that serves as an antifungal antibiotic and selectively inhibits the class I and II mammalian HDACs [8]. The diverse functions of HDAC inhibitors in mediating anticancer activities at different cellular levels, including histone acetylation-independent regulation, have been reported, although the causative mechanism remains undefined [9]. As HDAC inhibitors are well tolerated by cancer patients with manageable side effects, many of them, including those with broad-spectrum non-selective activity, are under preclinical and clinical evaluation. Although some HDAC inhibitors are successful in treating hematologic malignancies, their use in solid tumors remains controversial [9, 10]. A better comprehension of HDACs in cancer will give us a mechanistic-based rationale for the clinical use of HDAC inhibitors as anticancer agents [11].

Head and neck squamous cell carcinoma (HNSCC), arising mainly in the oral cavity, larynx, and pharynx, is the sixth most prevalent cancer and one of the most aggressive malignancies worldwide [12, 13]. HNSCC cells are primarily hypoacetylated as evidenced by lower levels of Acetyl-H3 compared to control oral keratinocytes, and tumor microenvironmental cues (e.g.*,* endothelial cell-secreted factors) can induce acetylation in HNSCC cells [14]. These findings suggest that use of HDAC inhibitors can represent a novel strategy for anti-HNSCC.

Here, we use TSA and PXD101 to demonstrate that HDAC inhibitors have the potential to induce repression of HNSCC aggressiveness and to inactivate ADP-ribosylation factor 1 (Arf1), a small GTPase involved in regulation of membrane trafficking pathways [15–17]. Further studies revealed the activity of Arf1 was much higher in metastatic HNSCC cells than cells derived from the primary sites, and HDAC inhibitors induced protein degradation of epidermal growth factor receptor (EGFR), which consequently suppressed Arf1 activation in HNSCC cells. Our novel findings provide precise mechanistic insights into action of HDAC inhibitors by exploring the previously unrecognized function in interrupting the EGFR-Arf1 complex in HNSCC progression, which provide the rationale for further clinical applications of this strategy in patients with HNSCC.

## Methods

### Cell lines and standard assays

HNSCC metastatic cell lines HN4, HN12, HN30 and HN31 were a gift from Dr. W. Andrew Yeudall [13]. All cells were maintained in Dulbecco’s modified Eagle’s medium (DMEM) containing 10% fetal bovine serum at 37 °C in a humidified incubator supplied with 5% CO_2_. Arf1 activation was determined by the glutathione resin-bound GST-GGA3-PBD fusion protein as described previously [15, 17]. Western blotting, wound closure assays, and cell proliferation assays were carried out as described previously [13, 18, 19].

### Reagents, constructs and antibodies

TSA, PXD101 and erlotinib were purchased from Selleckchem (Houston, TX). MG132 and recombinant human EGF were purchased from Sigma-Aldrich (St Louis, MO) and ProSpecBio (East Brunswick, NJ), respectively. The Arf1 dominant negative and constitutively active constructs pcDNA3-HA-Arf1 DN-T31 N (Arf1DN) and pcDNA3-HA-Arf1-ActQ71L (Arf1CA) were purchased from Addgene (Plasmid #10833 and #10832). Antibodies that recognize acetyl-Histone H3 (Lys9/Lys14), acetyl-Histone H4 (Lys8), p-AKT (Ser473), AKT, p-ERK1/2 (Thr202/Tyr204), ERK1/2, p-STAT3 (Tyr705), STAT3, p-Src (Tyr416), Src, p-EGFR (Tyr845), EGFR, p-ErbB2 (Tyr1221/1222), ErbB2, p-ErbB3 (Tyr1289) and ErbB3, were purchased from Cell Signaling Technology (Beverly, MA). β-actin and PY20 antibodies were purchased from Sigma-Aldrich (St Louis, MO). CellTiter 96® AQueous One Solution Cell Proliferation Assay (MTS) Kit was obtained from Promega (Madison, MI).

### HDAC activity assay

HDAC activity was measured with the fluorometric HDAC Activity Assay kit (Abcam, Cambridge, MA) according to the manufacturer’s instruction. Briefly, the cell lysates with or without TSA treatment were sonicated, cleared, and incubated with assay buffer containing the HDAC substrate [Boc-Lys(Ac)-AMC] for 30 min at 37 °C. The reaction was terminated, and the fluorescence intensity was measured in a fluorescence plate reader (Ex/Em = 350–380/440–460 nm).

### Phospho-receptor tyrosine kinase (RTK) profiling

The Proteome Profiler Human Phospho-RTK Array Kit (R&D Systems, Minneapolis, MN) was used to determine phosphor-RTK profiling according to the manufacturer’s instructions. Briefly, a total of 500 μg fresh protein was diluted and incubated overnight with nitrocellulose membranes dotted with duplicate spots for 42 anti-RTK and control antibodies. Bound phospho-RTKs were detected with a pan antiphosphotyrosine antibody conjugated to horseradish peroxidase using ECL reagents from Bio-Rad (Hercules, CA).

### Immunoprecipitation (IP)

In vitro protein-protein interactions were assessed by IP as described previously [20, 21]. Briefly, a total of 500 μg of cell lysate was used and diluted in 500 μl IP lysis buffer (containing 20 mM Tris·HCl-pH 8.0, 137 mM NaCl, 2 mM EDTA, 1% Nonidet P-40, and a mixture of protease and phosphatase inhibitors) with the corresponding antibodies. The lysate was incubated with gentle rotation overnight at 4 °C, and reaction mixtures were incubated with Protein A/G Sepharose® (Abcam, Cambridge, MA) for another 4 h at 4 °C. The immunoprecipitated proteins were washed three times with a buffer (containing 10 mM Tris ·HCl-pH 7.4, 150 mM NaCl, 1 mM EDTA, 1 mM EGTA, and 1% Triton X-100) followed by SDS-PAGE analysis with the indicated antibodies.

### Statistical analysis

Statistical differences were calculated using two-tailed Student’s t test. The data were expressed as means ± SD from triplicate experiments unless other indicated, and a *p*-value less than 0.05 was taken as statistically significant.

## Results

### TSA induces histone acetylation and suppresses proliferation, migration and invasion of HNSCC cells

**Fig. 1:**
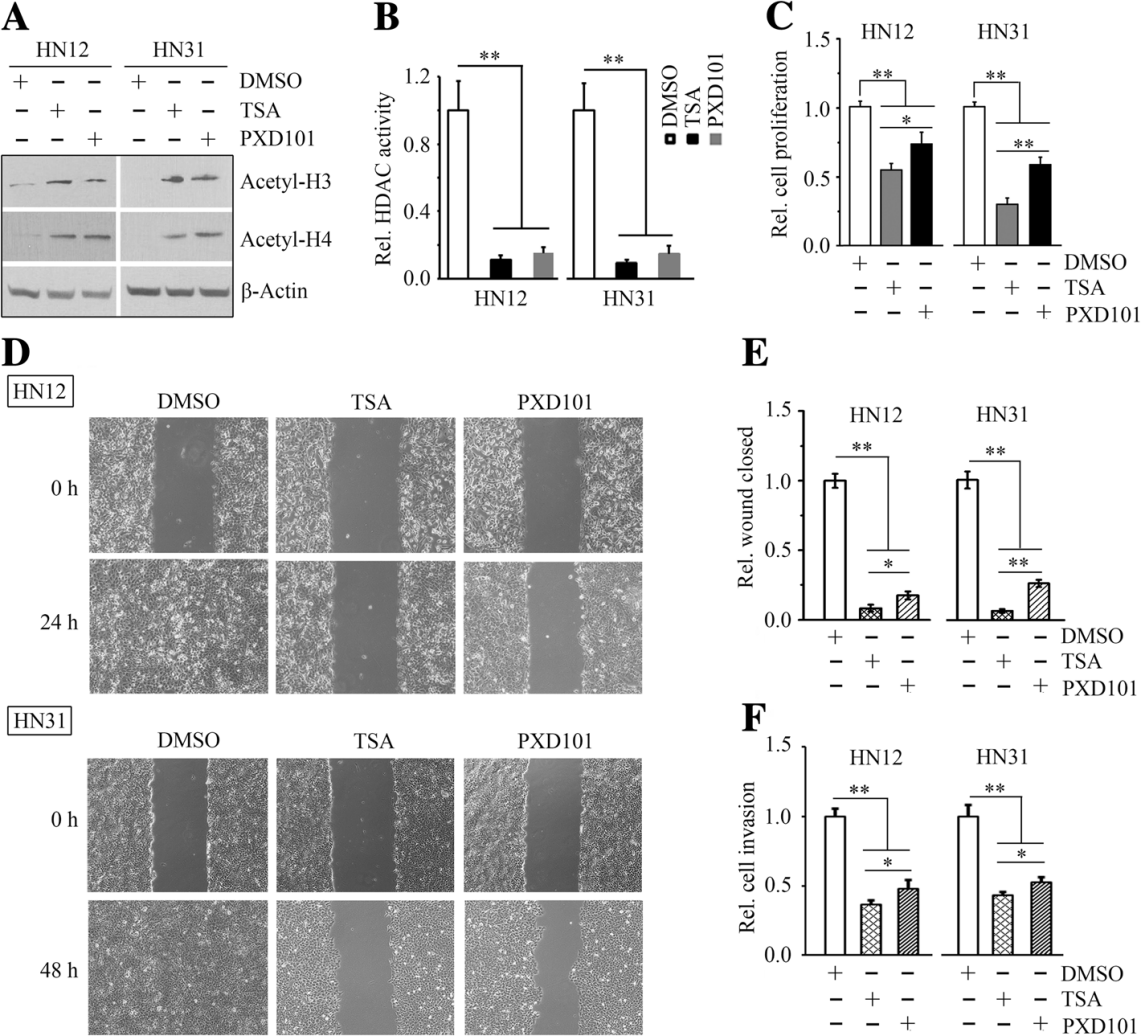
Either TSA or PXD101 induces acetylation of histones and inhibits proliferation, migration and invasion of HNSCC cells. **a** The effects of TSA and PXD101 on acetylation of histones H3 and H4 determined by Western blotting within 4 h after drug treatment. **b** The effects of TSA and PXD101 on regulation of HDAC activity measured by Abcam’s HDAC Activity Assay Kit (Fluorometric). **c** The effects of TSA and PXD101 on HNSCC cell proliferation measured by MTS assays within 72 h after drug treatment. **d**, **e** The effects of TSA and PXD101 on HNSCC cell migration measured by wound closure assays. Representative images were shown in (**d**) and quantitative data from three independent experiments were shown in (**e**). **f** The effects of TSA and PXD101 on HNSCC cell invasion measured by Borden chambers pre-coated with Matrigel. In this study, 5 μM TSA and PXD101 were used to treat HN12 and HN31 cells. *p < 0.05; **p < 0.01

Given that histones in mammalian cells treated with HDAC inhibitors are acetylated to an unusually high extent, we sought to determine the changes in histone acetylation levels in HNSCC cells in the presence or absence of TSA. Western blotting analysis revealed that TSA at 5 μM induced pronounced histone H3 and H4 hyperacetylation (Fig. 1a), and suppressed HDAC activity (Fig. 1b) in both HN12 and HN31 cells. MTS, wound healing and Borden chamber assays further showed that TSA has the strong potential against HNSCC cells, as evidenced by decreased proliferation, migration and invasion of HN12 and HN31 cells (Fig. 1c-1e). PXD101, another potent HDAC inhibitor, was also used in this study, which showed the similar effects observed from TSA treatment (Fig. 1a-1f). However, compared with PXD101, TSA at the same dose has superior anticancer activity in HNSCC cells (Fig. 1c-1f).

### TSA inhibits RTK phosphorylation in HNSCC cells

**Fig. 2:**
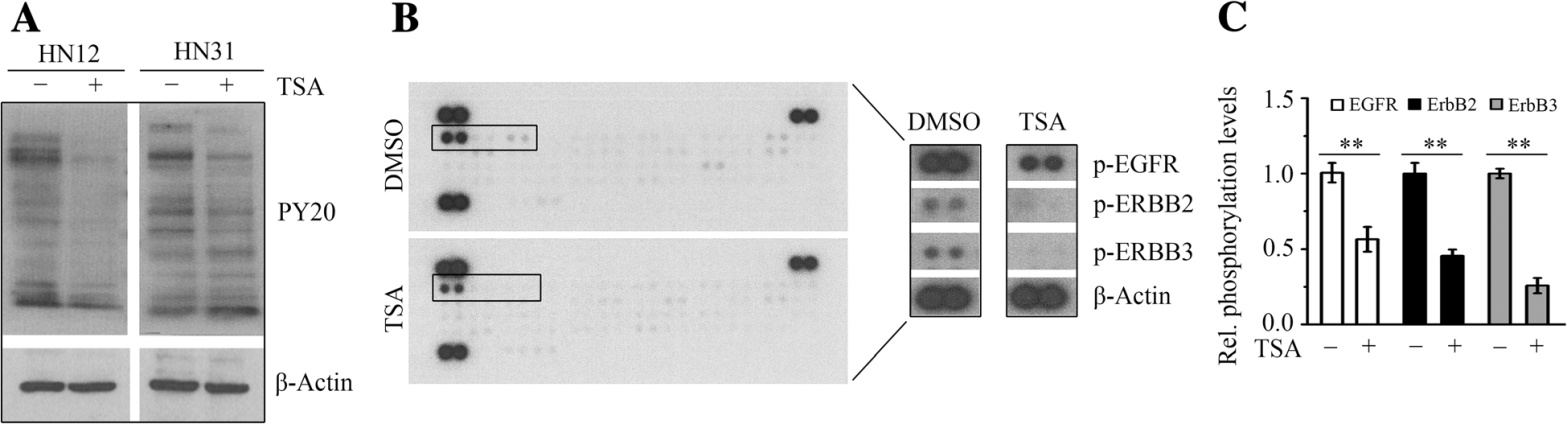
TSA downregulates the phosphorylation levels of RTKs in HNSCC cells. **a** The effect of TSA on global tyrosine phosphorylation determined by Western blotting with anti-phospho-tyrosine PY20 antibody. **b**, **c** The effect of TSA on the tyrosine phosphorylation levels of EGFR family members determined by Human Phospho-RTK array. Representative images were shown in (**b**) and quantitative data from three independent experiments were shown in (**c**). In this study, 5 μM TSA was used to treat HN12 and HN31 cells. **p < 0.01

RTK-regulated pathways play key roles in various facets of cancer progression [22, 23]. To better understand the mechanisms of TSA action in HNSCC cells, we first determined the extent and duration of tyrosine phosphorylation in the presence or absence of TSA. TSA at 5 μM impaired global tyrosine phosphorylation in HN12 and HN31 cells (Fig. 2a). We then determined the phosphorylation status of RTKs following TSA treatment using Proteome Profiler Human Phospho-RTK Array Kit, which showed that TSA reduced the phosphorylation levels of most RTKs examined in this study (Fig. 2b). Among them, the phosphorylation levels of EGFR were reduced to half in HN12 cells exposed to TSA (Fig. 2b and c). Other EGFR family members, ERBB2 and ERBB3, their phosphorylation was also markedly downregulated upon TSA treatment (Fig. 2b and c).

### TSA induces EGFR degradation through the ubiquitin-proteasome pathway in HNSCC cells

**Fig. 3:**
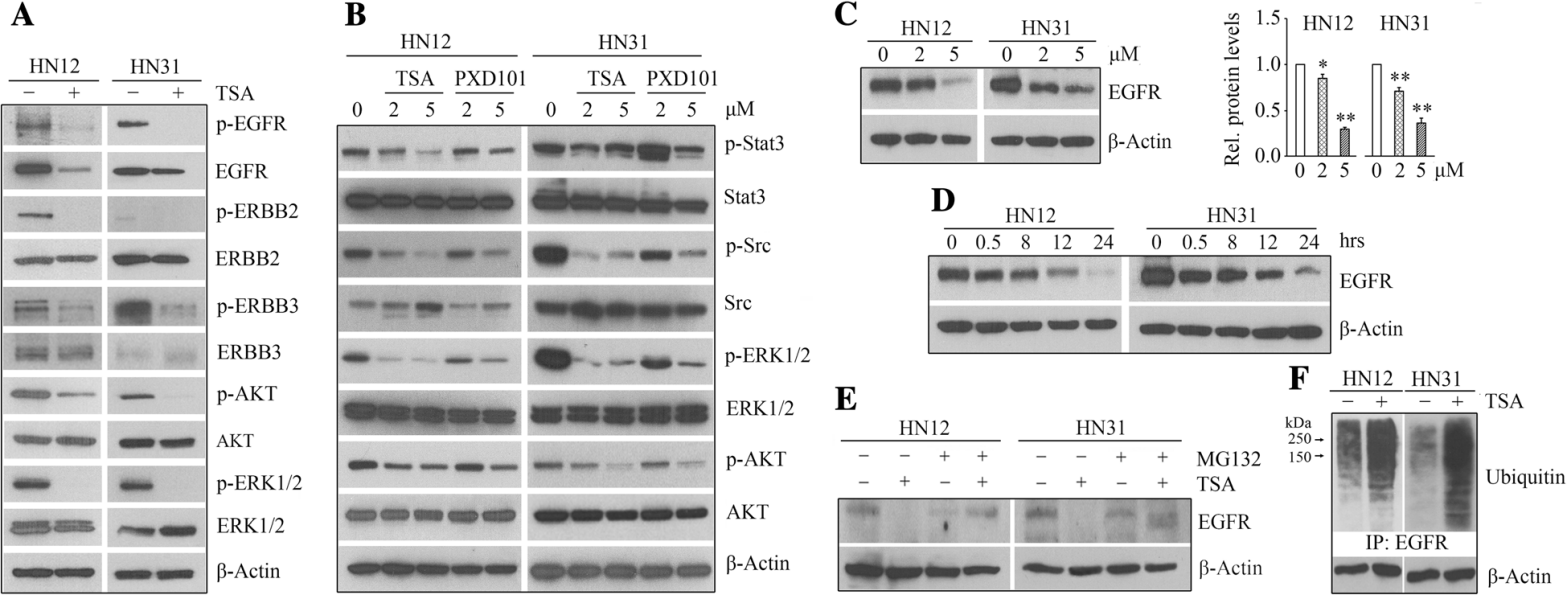
TSA downregulates EGFR through the proteasomal degradation pathway in HNSCC cells. **a** The suppressive effects of TSA on EGFR family proteins confirmed by Western blotting. **b** The effects of TSA and PXD101 on regulation of EGFR-meditated signaling pathways determined by Western blotting. **c**, **d** The dose- and time-dependent effect of TSA on EGFR determined by Western blotting. In **c**, representative images and quantitative data from three independent experiments were shown in the left and right panels, respectively. **e** The protein levels of EGFR in the presence of 5 μM TSA alone, 10 μM MG132 alone, or in combination. **f** The effect of TSA on EGFR ubiquitination. Cells were pretreated with 10 μM MG132 for 4 h before TSA treatment. The cell lysates were IP with anti-EGFR antibody and immunoblotted with anti-Ubiquitin antibody. In these assays, TSA and PXD101 were used to treat HN12 and HN31 cells. *p < 0.05; **p < 0.01

To determine whether TSA affects total protein amount of EGFR family members, HN12 and HN31 cells were incubated in the presence or absence of TSA for 24 h, and cell lysates were collected for Western blotting. This analysis confirmed the findings obtained from Phospho-RTK Array that TSA suppressed the phospho-activation of EGFR family proteins (Fig. 3a). Most importantly, TSA not only inhibited EGFR phosphorylation, but also induced repression of EGFR total protein amount in both HN12 and HN31 cells (Fig. 3a). There were no changes in ERBB2 and ERBB3 protein levels in the presence or absence of TSA (Fig. 3a). Not surprisingly, EGFR-mediated downstream signaling molecules, including STAT3, Src, ERK1/2 and AKT, their phosphorylation levels were downregulated following TSA treatment (Fig. 3a and b). PXD101 had a similar role in inhibition of EGFR signaling, but it was less efficient compared with TSA at the same concentrations (Fig. 3b). To further elucidate the mechanism of TSA-induced EGFR inhibition, HN12 and HN31 cells were treated with different concentrations of TSA, which showed that TSA dose-dependently inhibited EGFR at the protein level (Fig. 3c). Moreover, TSA at 5 μM exerted a suppressive effect on EGFR at 30 min (Fig. 3d). A more dramatic reduction in EGFR protein amount was seen when these cells were treated with TSA for 12 h, and it reduced to less than 10% at 24 h after treatment (Fig. 3d). Real-time RT-PCR showed that no changes in EGFR mRNA levels upon TSA treatment (data not shown). We next used the proteasome inhibitor MG132 in combination with TSA to determine whether EGFR can be degraded through the proteasome pathway following TSA treatment. Consistently, treatment with TSA alone resulted in reduced EGFR protein levels (Fig. 3e). However, addition of MG132 to TSA led to a significant increase in EGFR protein amount in both HN12 and HN31 cells compared with TSA treatment alone (Fig. 3e). To determine whether EGFR undergoes polyubiquitination in TSA treatment, MG132-pretreated HN12 and HN31 cells in the presence or absence of TSA were immunoprecipitated with anti-EGFR antibody. The EGFR immunocomplex displayed an increase in total ubiquitination in the presence of TSA (Fig. 3f), suggesting that ubiquitin-proteasome-dependent degradation is the main machinery involved in TSA-induced EGFR repression in HNSCC cells.

### TSA inactivates Arf1 to suppress invasion of HNSCC cells

**Fig. 4:**
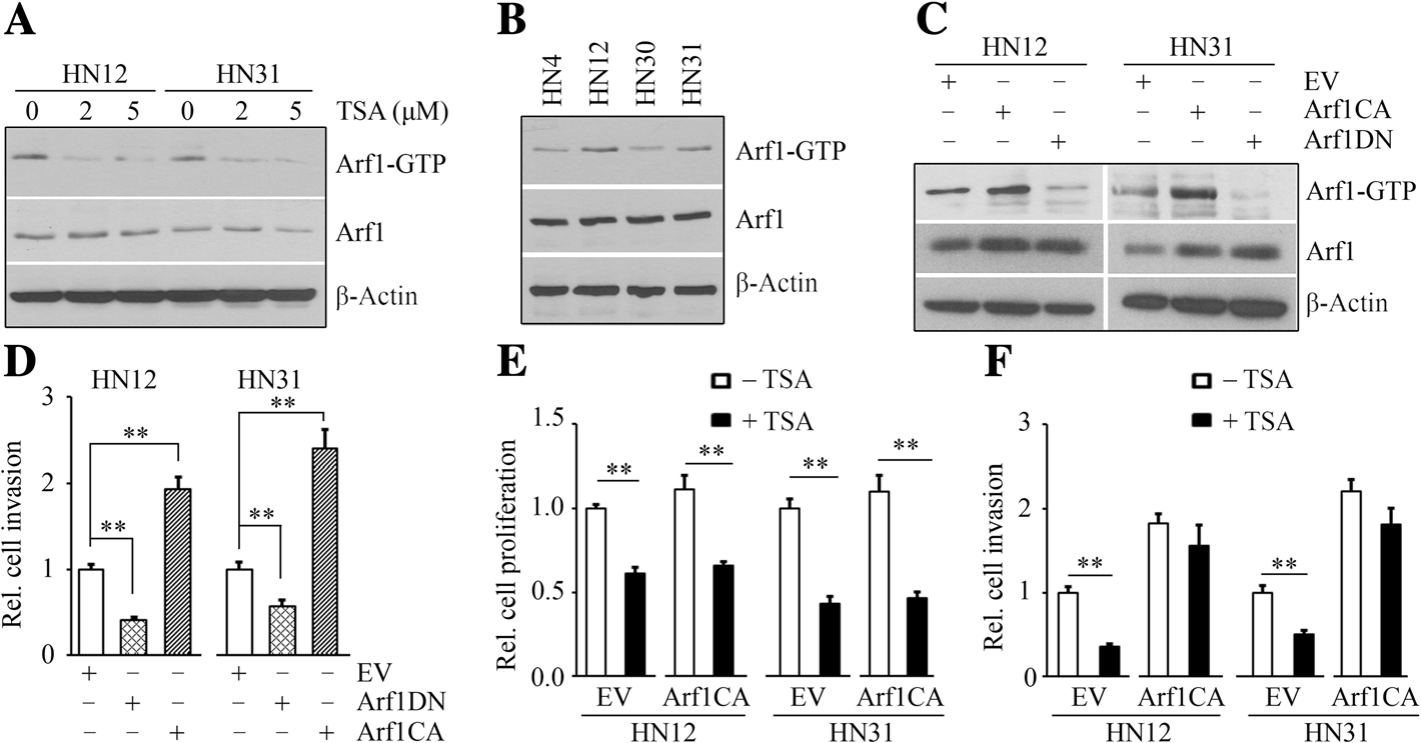
TSA inhibits cell invasion through inactivating Arf1 in HNSCC cells. **a** The effect of TSA on Arf1 activation measured by GST-VHS-GAT pulldown assays. **b** Arf1 protein levels and activity in various HNSCC cells determined by Western blotting. **c** The effects of expression of Arf1DN and Arf1CA on Arf1 activation measured by GST-VHS-GAT pulldown assays. **d** The effect of expression of Arf1DN and Arf1CA on HNSCC cell invasion measured by Borden chambers pre-coated with Matrigel. **e**, **f** The effect of expression of Arf1CA on HNSCC cell proliferation (**e**) and invasion (**f**) in the presence or absence of 5 μM TSA. Cell proliferation was measured by MTS within 72 h after drug treatment, and cell invasion was determined by Borden chambers pre-coated with Matrigel. EV: empty vector; Arf1CA: the constitutively active mutant of Arf1; Arf1DN: the dominant negative mutant of Arf1; **p < 0.01

Arf1 activation can be regulated by RTKs [24]. As TSA can induce repression of RTKs in HNSCC cells (Fig. 2), we determined whether TSA has the ability to regulate Arf1 function. TSA, at 2 or 5 μM, did not alter total Arf1 protein levels in both HN12 and HN31 cells (Fig. 4a). We thus performed pull-down experiments with a GST-GGA3-PBD fusion protein to determine Arf1 activation under the influence of TSA. Interestingly, the levels of active GTP-bound Arf1 were strongly inhibited upon TSA treatment (Fig. 4a). Arf1 has been reported to be critical for breast cancer cell invasion and metastasis [15, 25, 26]. To determine whether this is true in HNSCC cells, we examined the expression pattern of Arf1 in two cell line pairs (HN4-HN12 and HN30-HN31). HN4 and HN30 were derived from primary lesions in the base of tongue and pharynx, respectively, and HN12 and HN31 were derived from lymph-node metastatic lesions belonging to the same patients [27]. No notable difference in Arf1 protein levels was seen among these cells (Fig. 4b). The levels of active GTP-bound Arf1 were much stronger in metastatic HN12 and HN31 cells compared with their paired non-metastatic HN4 and HN30 cells (Fig. 4b), supporting the critical role of Arf1 activation in HNSCC metastasis. We then determined the importance of Arf1 activation in HNSCC cells. Arf1CA and Arf1DN were overexpressed in HN12 and HN31 cells, leading to increased or decreased Arf1 activation, respectively (Fig. 4c). These alterations in Arf1 activation levels did not affect HNSCC cell proliferation (Additional file 1: Figure S1). However, inactivation of Arf1 by transfection with Arf1DN significantly suppressed invasion of HNSCC cells and activation of Arf1 by transfection with Arf1CA counteracted this effect (Fig. 4d). These observations indicate that GTP-bound Arf1 is critical for HNSCC cell invasion. As TSA can induce repression of HNSCC cell invasion, we determined the consequences of Arf1CA transfection in the presence of TSA. Consistently, TSA at 5 μM decreased proliferation rate and invasive potential in HN12 and HN31 cells (Fig. 4e and f). No significant changes in cell proliferation were observed from TSA-treated cells transfected with Arf1CA or not (Fig. 4e). However, increased Arf1 activation attenuated TSA-induced inhibition of invasion in HN12 cells (Fig. 4f). The similar tendency was observed in HN31 cells when transfected with Arf1CA in the presence or absence of TSA (Fig. 4e and f). These observations indicate that TSA exhibits a suppressive effect on HNSCC cell invasion, at least in part, through inactivating Arf1.

### Arf1 activation is upregulated by binding to phospho-EGFR in HNSCC cells

**Fig. 5:**
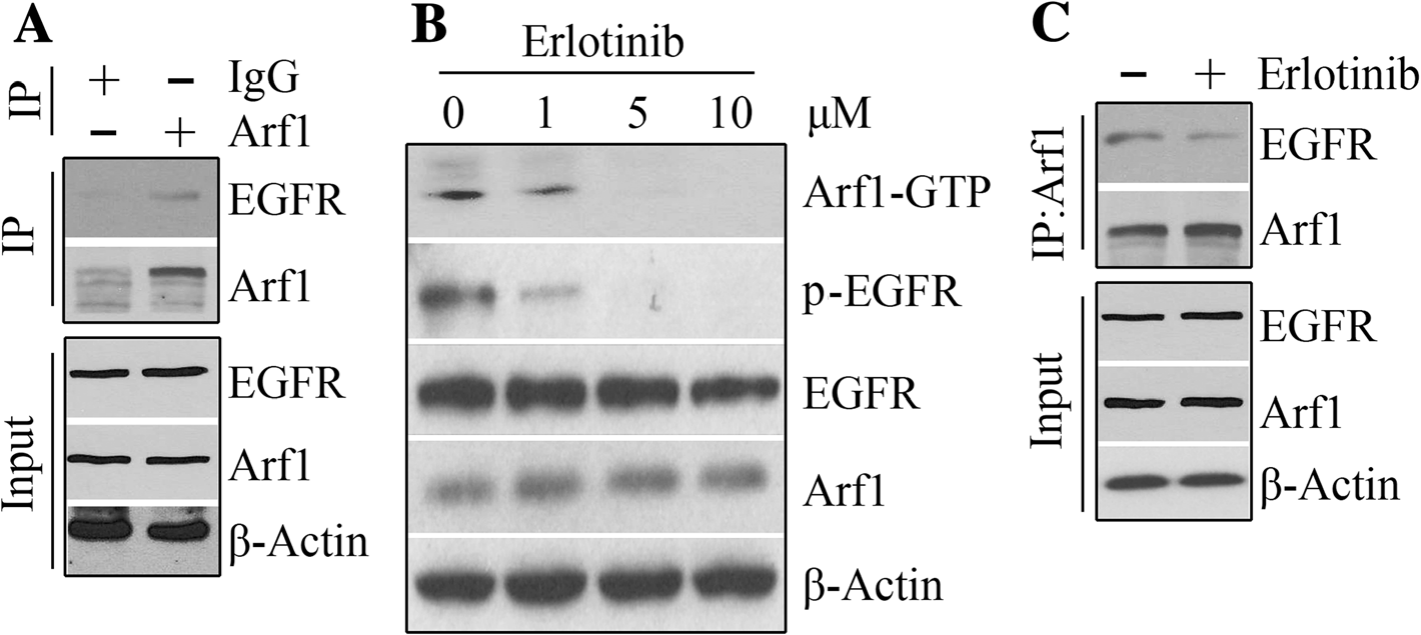
Phospho-EGFR binds to Arf1 in HNSCC cells. **a** The EGFR-Arf1 binding in HN12 cells determined by IP. Preimmune IgG was used as a negative control. **b** The effect of erlotinib on Arf1 activation determined by Western blotting. **c** The effect of 5 μM erlotinib on the engagement of Arf1 in the EGFR immunocomplex determined by IP

EGFR is the most important RTK overexpressed in up to 90% of HNSCC compared with levels in normal mucosa, where expression levels correlate with decreased survival, independent of therapy [28–31]. To determine the functional interaction between EGFR and Arf1 in HNSCC cells, we assessed their protein interaction. IP results from HN12 cells showed that Arf1 was one of EGFR interactors (Fig. 5a). To determine whether EGFR regulates Arf1 function in HNSCC cells, the small-molecule EGFR inhibitor erlotinib was used to treat HN12 cells. Twenty-four hours after treatment, erlotinib dose-dependently induced reduction in Arf1 activation without affecting total protein amount of Arf1 (Fig. 5b), and this reduction was associated with decreased EGFR phosphorylation levels (Fig. 5b). The total EGFR levels were only decreased at high dose of 10 μM erlotinib (Fig. 5b). IP was then used to assess the binding ability of Arf1 to EGFR in the presence of 5 μM erlotinib, which revealed decreased engagement of Arf1 in the EGFR immunocomplex compared with non-treatment (Fig. 5c). These findings suggest that phosphorylated EGFR binds to and activates Arf1 in HNSCC cells.

### TSA inhibits EGF-induced HNSCC cell invasion through suppressing the EGFR-Arf1 signaling complex

**Fig. 6:**
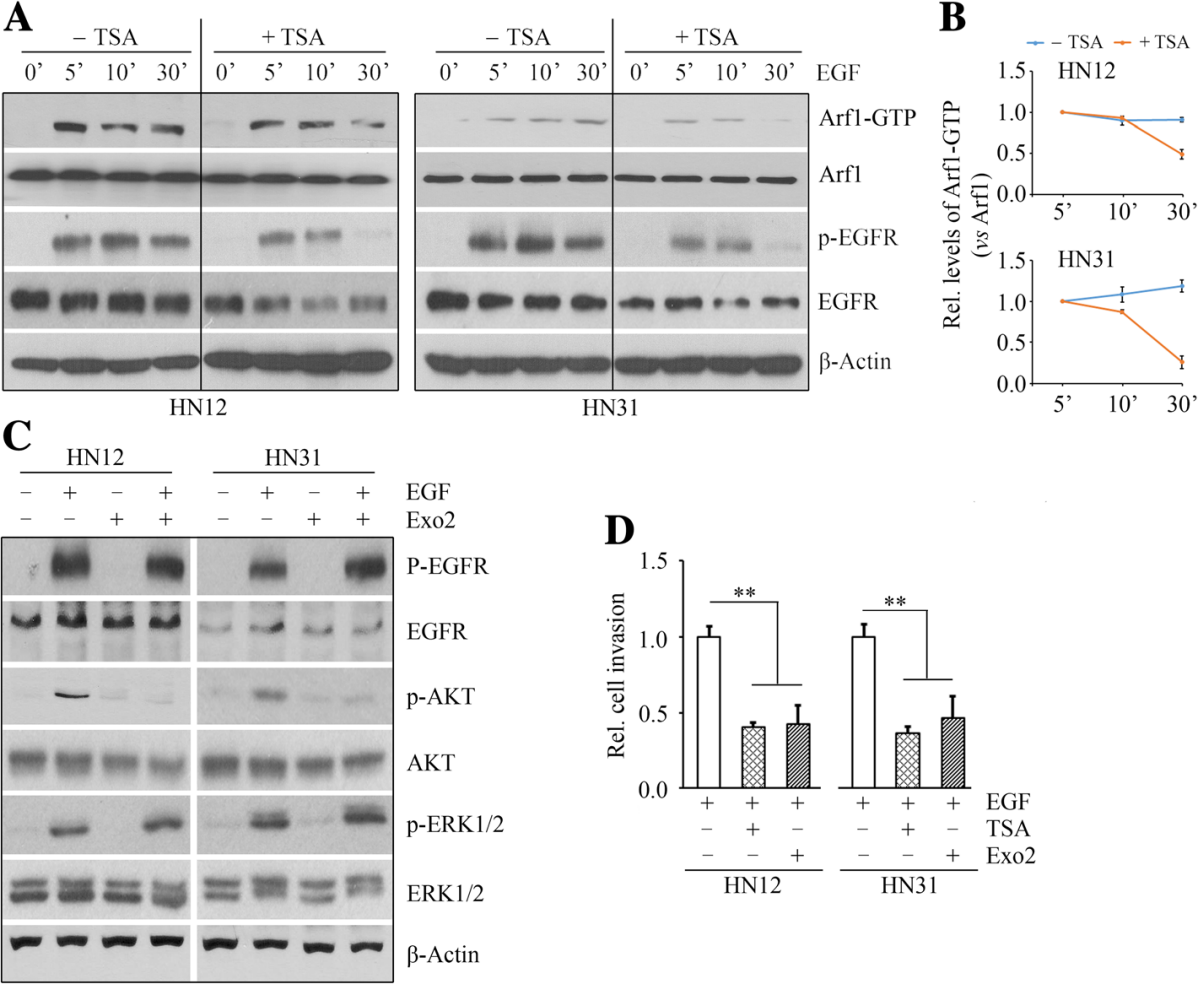
TSA inhibits EGF-induced HNSCC cell invasion through suppressing the EGFR-Arf1 complex. **a**, **b** The effect of TSA on EGF-induced Arf1 activation. Representative images were shown in (**a**) and quantitative data from three independent experiments were shown in (**b**). **c** The effect of the Arf1 inhibitor Exo2 on EGF-induced signaling pathways. **d** The effects of TSA and Exo2 on EGF-induced HNSCC cell invasion measured by Borden chambers pre-coated with Matrigel. In these assays, EGF at 50 ng/ml, TSA at 5 μM, or Exo2 at 20 μM was used to treat HN12 and HN31 cells. **p < 0.01

To better define the specific role of EGF signaling in Arf1 activation, serum-starved HNSCC cells were treated with 50 ng/ml human recombinant EGF for different times. EGF-induced Arf1 activation was accompanied by an associated increase in EGFR phosphorylation in HN12 cells within 5 min (Fig. 6a). The same was true for HN31 cells upon EGF stimulation (Fig. 6a and b). However, Arf1 activation triggered by EGF was significantly blocked when EGFR phosphorylation was impaired by 5 μM TSA at 30 min post treatment (Fig. 6a and b), indicating that TSA-induced inactivation of Arf1 is through EGFR blockade. To determine the contribution of Arf1 to EGF signaling, HNSCC cells were treated with EGF in the presence or absence of TSA or the Arf1 inhibitor Exo2. Interestingly, Exo2 markedly attenuated EGF-induced AKT activation, but it played no role in regulating ERK1/2 signaling upregulated by EGF (Fig. 6c). Moreover, either TSA or Exo2 significantly suppressed EGF-induced cell invasion (Fig. 6d). These data indicate that Arf1 activation plays an essential role in HNSCC cell invasion driven by EGFR.

## Discussion

Dysregulation of HDACs and aberrant chromatin acetylation and deacetylation have been implicated in the pathogenesis of various diseases, including cancer. HDAC inhibitors, such as TSA, exhibit their anticancer activity by promoting the acetylation of histones, leading to uncoiling of chromatin and activation of a variety of genes involved in the regulation of oncogenesis. However, the therapeutic effects of HDAC inhibitors on cancer invasion and metastasis and the underlying action mechanisms are rarely reported. The present study reveals that suppression of HNSCC cell migration and invasion upon TSA treatment is partially through its mediated downregulation of the EGFR-Arf1 signaling. These promising findings aide in understanding the complex set of molecular events in HNSCC cells in response to HDAC inhibitors. As there is an increasing interest in using HDAC inhibitors to treat various cancers in the clinic, the knowledge gained from this study would be significantly beneficial for the development of new rational HDAC-targeted anticancer modalities.

Overexpression of EGFR is a significant finding in cancer, particularly in HNSCC, where it is positively associated with a poor prognosis of patients [32, 33]. The basic mechanism of EGFR activation and the role of EGFR signaling in cancer progression, has been well studied. Targeting the EGFR signaling pathway by the various anti-EGFR therapeutic agents currently represents a promising treatment strategy for epithelial cancers. Interestingly, EGFR expression and function have been reported to be regulated by HDACs [34–36]. These studies include HDAC6 inhibition-induced EGFR endocytic trafficking and degradation in renal epithelial cells [34] and HDAC3-CAGE axis-mediated EGFR activation in fibroblast melanoma cells [37]. On the other hand, the regulation of HDACs is also influenced by EGFR signaling. A good example is that EGFR suppresses Runx2 expression through upregulating the expression of HDAC4/6 [38]. Our work expands the observation that HDAC inhibitors could serve as a single agent to block EGFR, which may or may not directly rely on the activity of HDACs in HNSCC cells.

Arf1 is one of Arf family GTPases and acts as a crucial regulator for vesicular vesicle trafficking. Disrupting its function blocks membrane protein recycling and translocation of proteins from trans-Golgi network to plasma membrane [15–17]. Amplification is the predominant type of alteration for Arf1 in breast cancer cells, and its frequency was much higher than other Arf family members [15]. Here, we show for the first time that Arf1 plays a critical role in promoting migration and invasion in HNSCC cells. Mechanistic study indicates that GTP-bound Arf1 active form binds to EGFR in HNSCC cells, and inhibition of EGFR by erlotinib dramatically impairs Arf1 activation. Most importantly, HDAC inhibitors, such as TSA, can induce the proteasome degradation of EGFR, which in turn inactivating Arf1, leading to suppression of tumor-promoting activity. Interestingly, Arf1 is further overexpressed in highly invasive breast cancer cells compared with non-invasive breast cancer cells, and its expression levels are strongly associated with poor survival of breast cancer patients, which may be also attributed to the involvement of the EGFR-Arf1 complex in the progression of cell invasion and metastasis [15, 25, 39].

The best therapeutic regimen is to simultaneously target tumorigenesis and metastasis in order to prolong life of cancer patients. The treatment of HDAC inhibitors has been observed to augment cell migration and metastasis in human breast, gastric, liver, and lung cancer, which significantly ruins their therapeutic efficacy [40]. However, this effect mediated by HDAC inhibitors are cancer-type dependent as our observations clearly delineate that HDAC inhibitors can inhibit both proliferation and invasion in HNSCC cells. These obtained findings are consistently supported by the other study showing that inhibition of HDACs by TSA disrupts the accumulation of cancer stem cells and paradoxically induces epithelial-mesenchymal transition (EMT) of HNSCC cells [14].

Although the critical role of EGFR-Arf1 in HDAC-targeted treatment has been demonstrated in culture HNSCC cells, prospective studies are warranted to provide evidence showing the difference of EGFR-Arf1 activity in clinical metastatic and non-metastatic HNSCC samples and their correlation with the therapeutic efficacy of HDAC inhibitors. Currently, determination of Arf1 activity in clinical samples is challenging due to the lack of an applicable method to measure the levels of Arf1 active form in solid tissues. Therefore, a vigorous research effort is needed to seek a feasible way to examine EGFR-Arf1 activity in subsets of HNSCC patients in order to better assess or predict the clinical outcomes of HDAC inhibitors.

## Conclusions

Demonstrating that HDAC inhibitors inactivate Arf1 through destabilizing EGFR proteins in a histone acetylation-independent manner is very timely, which not only provides the importance of the EGFR-Arf1 complex in the development and progression of HNSCC, but also opens a promising therapeutic avenue for design of HDAC-targeted regimens to better treat HNSCC.

## Additional file

Additional file 1: **Figure S1.** The effects of expression of Arf1DN and Arf1CA on HNSCC cell proliferation measured by MTS. (DOCX 36 kb)

## Abbreviations

ADP: Adenosine diphosphate
Arf1: ADP-ribosylation factor 1
DMEM: Dulbecco’s modified Eagle’s medium
EGFR: Epidermal growth factor receptor
HDACs: Histone deacetylases
HNSCC: Head and Neck Squamous Cell Carcinoma
IP: Immunoprecipitation
PXD101: Belinostat
RTK: Receptor tyrosine kinases
SAHA: Vorinostat
TSA: Trichostatin A
