# Supplementary material for: Histone deacetylase inhibitors suppress aggressiveness of head and neck squamous cell carcinoma via histone acetylation-independent blockade of the EGFR-Arf1 axis
Source: J Exp Clin Cancer Res. 2019 Feb 18;38:84. doi: 10.1186/s13046-019-1080-8 (PMC6379952; doi:10.1186/s13046-019-1080-8)
Supplement: Supplementary file 1 — Figure S1. The effects of expression of Arf1DN and Arf1CA on HNSCC cell proliferation measured by MTS. (DOCX 36 kb) [file 13046_2019_1080_MOESM1_ESM.docx]

**Figure S1.** The effects of expression of Arf1DN and Arf1CA on HNSCC cell proliferation measured by MTS.
